# Trajectory tracking method based on the circulation of feasible path planning

**DOI:** 10.1371/journal.pone.0252542

**Published:** 2021-06-07

**Authors:** Yi Yu, Peng Han

**Affiliations:** 1 Department of General Aviation, Civil Aviation Management Institute of China, Beijing, China; 2 Zhe Jiang Key Laboratory of General Aviation Operation Technology, Jiande, China; 3 School of Air Traffic Management, Civil Aviation University of China, Tianjin, China; Tongii University, CHINA

## Abstract

The control method is the central point of the unmanned vehicles. As the core system to guarantee the properties of self-decision and trajectory tracking of the unmanned vehicles, a new kind of trajectory tracking method based on the circulation of feasible path planning for the unmanned vehicles are proposed in this article which considered the dynamics and kinematics characteristics of vehicles. The multi-trace-points cooperative trajectory tracking control strategy on the basis of the circulation of feasible path generation method is proposed and the lateral controller is designed for trajectory tracking. The process of feasible path generation is conducted once the tracking error exceeded. A simulation platform of the trajectory tracking simulation of unmanned vehicles is built considering the mechanical properties of system elements and the mechanical characteristics. Finally, the proposed trajectory tracking method is verified. The tracking error would be reduced to make sure the vehicles move along the pre-set virtual track.

## 1. Introduction

With the application of unmanned vehicles, the researches on trajectory tracking are very rich at this field. The unmanned vehicles are typically nonholonomic constraint system which have the characteristics of highly non-linear and complexity. Thus, it is difficult to propose the control strategy based on precise modeling. The commonly used tracking control strategy decouples the lateral and longitudinal motion to control the path following and velocity seperately.

The path following could be divided into preview control and compensation control. The preview control methods [1–3] calculated the target quantities to control the motion state of the unmanned vehicles based on the dynamics model and the kinematics model. The real time condition monitoring system should give a feedback of the actual lateral acceleration, yaw velocity, and sideslip angle, and so on. The controller made the difference between the calculated target and actual quantities decrease to approach the target path. Xia [4] proposed a novel approach combining the sliding mode control and extended state observer (ESO) for attitude control of a missile model. Tang [5] developed trajectory tracking and configuration stabilization for the vertical takeoff and landing (VTOL) aircraft which addressed global configuration stabilization with a strong input coupling using a smooth static state feedback. The compensation control method monitored the target trajectory and actual location of the vehicle. Then the actuators were controlled directly to move along the trajectory. The steering model, kinematics model and dynamics model were often used in the control system. The control method could also be classified based on the control strategy, such as PID method, optimum control, sliding-mode control, model predictive control, fuzzy control, and neural network control. Oreh [6] proposed a new desired articulation angle for directional control of the articulated vehicles. The proposed reference value tracking ensured that the rear end of the trailer unit closely followed the trajectory of the fifth wheel. Overcoming the methods mentioned above, several research efforts [2, 7–13] had been reported on dynamic analysis and active control systems development in articulated vehicles. The scopes of these researches were the maneuverability improvement and stability limits enhancement of the articulated vehicles. These methods applied in unmanned vehicles had obtained good results.

The development of these advanced technologies provided proper environments for the development of unmanned vehicles. On this background, a new trajectory tracking method based on the circulation of feasible path planning was proposed in this paper.

## 2. Kinematics model of unmanned vehicles

### 2.1. The expected position determination method

The location of the vehicle could be divided into two steps. The first step is to determine the position and attitude of the first element. Then, the location of the other elements could be decided according to the constraint conditions of the virtual track and articulate points. To ensure the vehicle moving along the track, the concepts of trace points are raised to guarantee to move along the track. The numbers of trace points should be subject to ensure the uniqueness property of position and attitude of each vehicle element. There are two basic factors for the trace points.

1. It is fixed in the carbody and moved with each element;
2. The trace points should always move along the track.

According to the two assumptions and the principle of rigid body kinematics, the third implicated assumption inferred that the velocity in trace points is along the tangential direction of the curvature.

**Fig 1:**
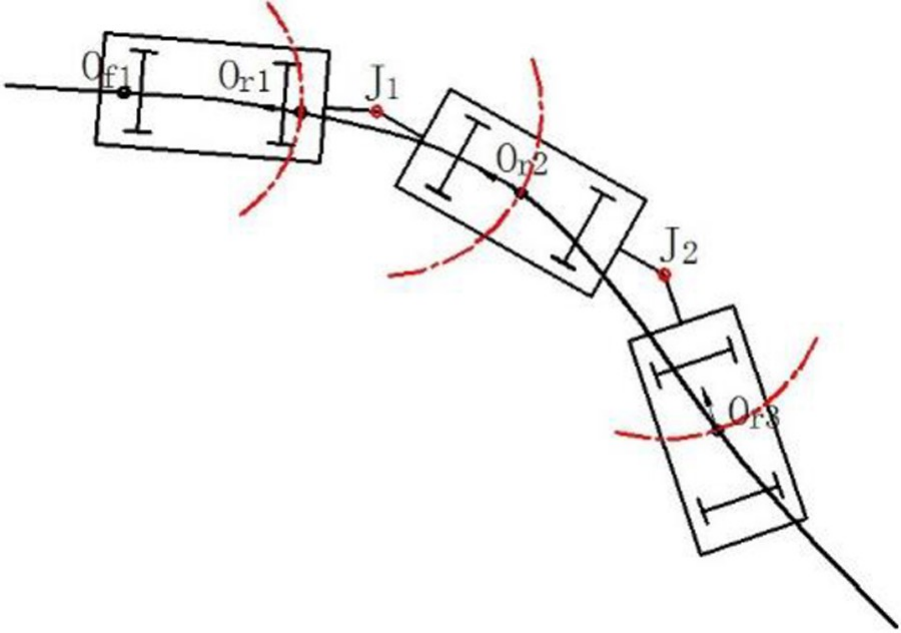
The expected position of each vehicle element along the track.

As is shown in Fig 1, the position and attitude of the first vehicle element should be confirmed uniquely by two trace points, and the following vehicles could be confirmed by only one trace point since the articulated point between two vehicles isalso used to locate. Thus, the expected position of each vehicle element along the virtual track is confirmed uniquely and definitely.

The trace points are labeled as *O*_*f*1_, *O*_*r*1_, *O*_*r*2_ and *O*_*r*3_. The absolute coordinates are described as $\left(x_{0_{f1}},y_{0_{f1}}\right)$ and $\left(x_{0_{r1}},y_{0_{r1}}\right)$. The attitude of each vehicle element and the position of each point in the vehicle could be described as follow.

$$\theta_{c1}=\tan^{-1}\left(\frac{y_{0_{r1}}-y_{0_{f1}}}{x_{0_{r1}}-x_{0_{f1}}}\right)$$ (1)

$$\left[\begin{array}{c} x_{J_{1}}\\ y_{J_{1}} \end{array}\right]=\left[\begin{array}{c} x_{0_{c1}}\\ y_{0_{c1}} \end{array}\right]+\left[\begin{array}{cc} \cos(\theta_{c1}) & -\sin(\theta_{c1})\\ \sin(\theta_{c1}) & \cos(\theta_{c1}) \end{array}\right]\left[\begin{array}{c} {x^{\prime }}_{J_{1}}\\ {y^{\prime }}_{J_{1}} \end{array}\right]$$ (2)

As well, *θ*_*c*2_, *θ*_*c*3_ and $\left(x_{0_{r3}},y_{0_{r3}}\right)$ could be determined in the same method.

### 2.2. Kinematic model of the vehicle

**Fig 2:**
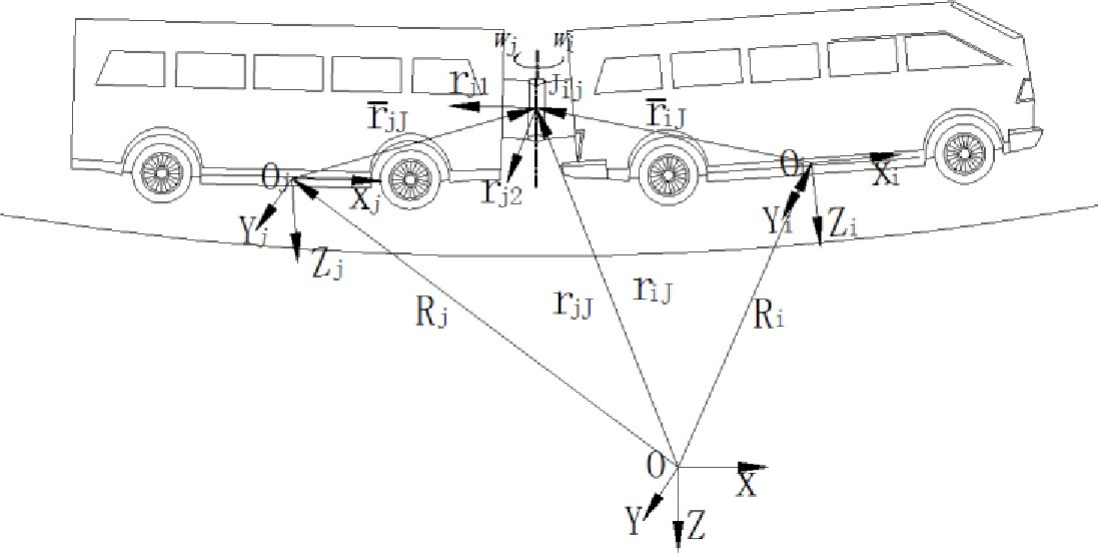
Kinematics constraints of the articulated mechanism.


As is shown in Fig 2, the kinematics constraints are acted between two vehicle elements. The moving coordinate system are denoted as *O*_*i*_*X*_*i*_*Y*_*i*_*Z*_*i*_ and *O*_*j*_*X*_*j*_*Y*_*j*_*Z*_*j*_. Taking the revolute joint as an example, three degrees of freedom including parallel horizontal and longitudinal motion are limited. Only the vertical rotational motion is retained. The revolution of two vehicle elements are recorded as ${\vec{\omega }}_{i}$ and ${\vec{\omega }}_{j}$. Two orthogonal vectors are selected in the second element and recorded as ${\vec{\omega }}_{j1}$ and ${\vec{\omega }}_{j2}$ separately. The equations of kinematics constraints in the hinge point are expressed as Eq (3).

$$C(q_{i},q_{j})=\left\{\begin{array}{c} r_{iJ}-r_{jJ}\\ \omega_{i}\cdot \omega_{j1}\\ \omega_{i}\cdot \omega_{j2} \end{array}\right\}=0$$ (3)

In which, $r_{iJ}$ and $r_{jJ}$ represent the position vector in the hinge point of the first and second vehicle element. Substituted the transformation matrix of the ground fixed coordinate and carbody following coordinate into Eq (3), we can get Eq (4).

$$\left\{\begin{array}{c} R_{i}+A_{i}{\vec{r}}_{iJ}-(R_{j}+A_{j}{\vec{r}}_{jJ})\\ A_{i}{\vec{\omega }}_{i}\cdot A_{j}{\vec{\omega }}_{j1}\\ A_{i}{\vec{\omega }}_{i}\cdot A_{j}{\vec{\omega }}_{j2} \end{array}\right\}=0$$ (4)

In the formula, ***R***_*i*_ and ***R***_*j*_ represent the position vector of the original point of carbody following coordinates. The Jacob matrix of the constraint equation is expressed as follow.

$${\dot{C}}_{q}(q_{i},q_{j})=\frac{\partial C}{\partial q}=\left[\begin{array}{cccc} I & -A_{i}{\bar{\bar{r}}}_{iJ}{\bar{G}}_{i} & -I & -A_{i}{\bar{\bar{r}}}_{jJ}{\bar{G}}_{j}\\ 0 & -A_{i}{\bar{\bar{\omega }}}_{i}{\bar{G}}_{i}\cdot A_{j}{\bar{\omega }}_{j1} & 0 & -A_{i}{\bar{\omega }}_{i}\cdot A_{j}{\bar{\bar{\omega }}}_{j1}{\bar{G}}_{j}\\ 0 & -A_{i}{\bar{\bar{\omega }}}_{i}{\bar{G}}_{i}\cdot A_{j}{\bar{\omega }}_{j2} & 0 & -A_{i}{\bar{\omega }}_{i}\cdot A_{j}{\bar{\bar{\omega }}}_{j2}{\bar{G}}_{j} \end{array}\right]=\left[C_{q_{i}}\;C_{q_{j}}\right]$$ (5)

**Fig 3:**
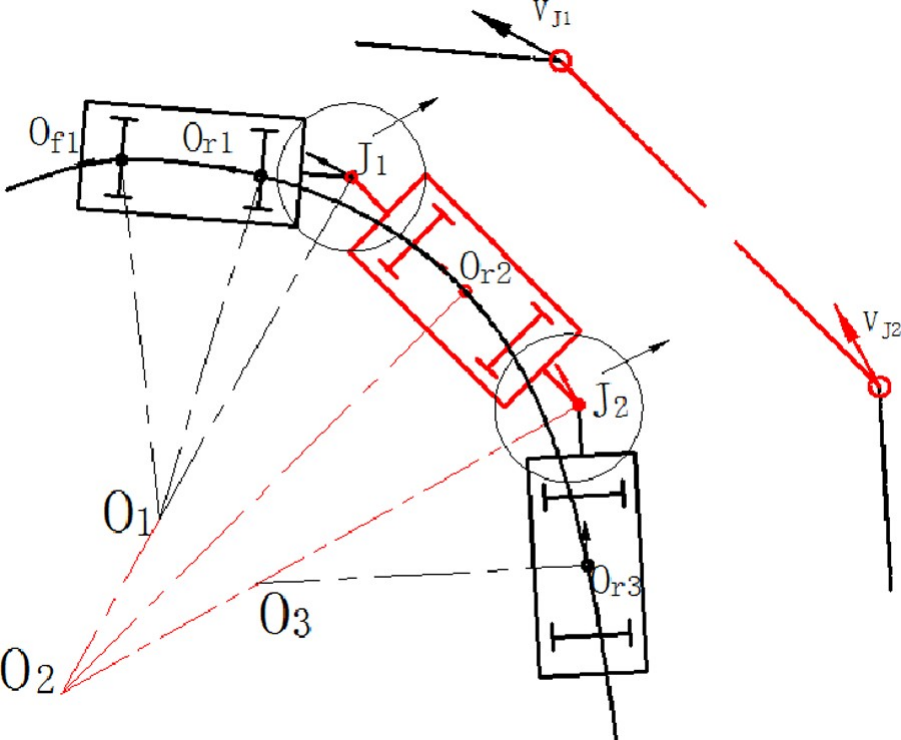
The kinematics model of the unmanned articulated vehicle.


As the pre-designation of the vehicle, all the vehicle elements are all-wheel drived. Fig 3 shows the instantaneous rotation center of each element and the velocity of the hinge joint. The instantaneous rotation center is decided by the velocity direction of the hinge joint and trace point. The velocity in the trace point is consistent with the carbody.

$$\frac{{\vec{v}}_{0_{r}}}{\left|v_{0_{r}}\right|}=\frac{\vec{x^{\prime }}}{\left|x^{\prime }\right|}$$ (6)

Assumed that the velocity of the trace point is the control objective, the velocity of each wheel could be calculated according to the kinematics model of one element of the vehicle. The velocity of the hinge joint could be calculated based on the first vehicle element. The velocity in point *J*_1_ is expressed as follow.

$$v_{J_{1}}=\frac{v_{0_{f1}}}{\left|0_{f1}O_{1}\right|}*\left|J_{1}O_{1}\right|=\frac{v_{0_{f1}}}{\sqrt{{\left(x_{0_{f1}}-x_{O_{1}}\right)}^{2}+{\left(y_{0_{f1}-}y_{O_{1}}\right)}^{2}}}*\sqrt{{\left(x_{o_{r2}}-x_{O_{1}}\right)}^{2}+{\left(y_{o_{r2}-}y_{O_{1}}\right)}^{2}}$$ (7)

In this way, the velocity of the wheels in the follow-up elements could be calculated.

## 3. Feasible path planning

**Fig 4:**
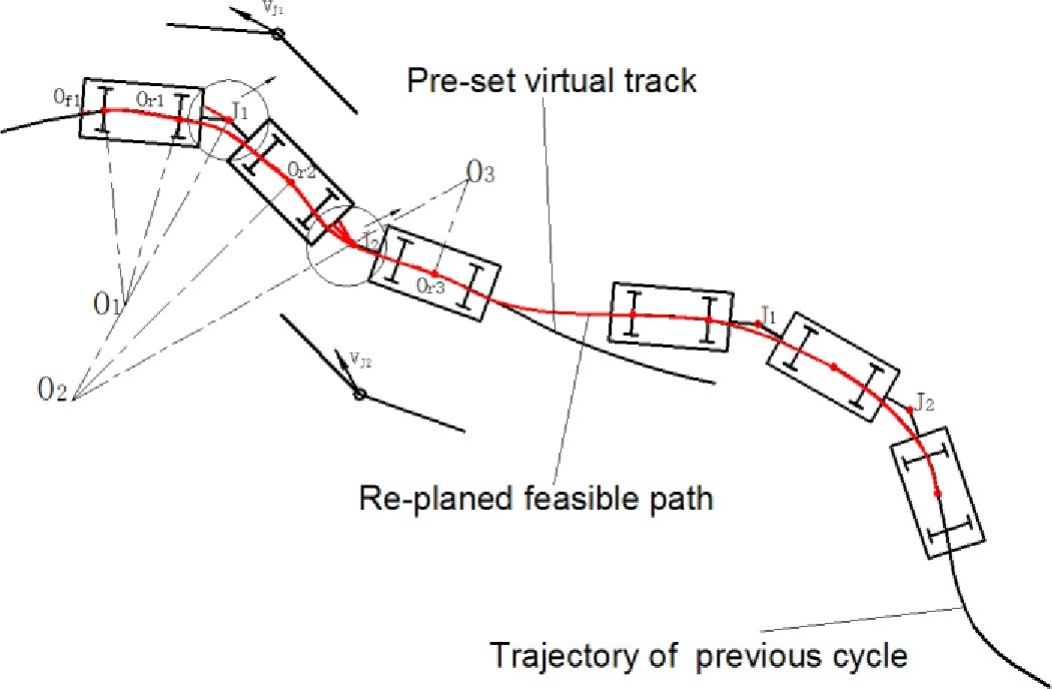
Feasible path planning.

The aim of feasible path planning is to generate a feasible path of the vehicle which satisfy the kinematics constraints, boundary conditions and the characteristic of actuators. As Fig 4 shows, the problem of path planning could be represented as the planning of a series of movement attitudes and gestures between the original state and the terminal state. The vehicle could operate according to the planning gestures to achieve the goal of path following.

The physical quantities which describe the motion of vehicle are labelled as set ***C***. The set contains the location coordinates of each trace point $\left(x_{O_{bm}},y_{O_{bm}}\right)$, the instantaneous turn center $\left(x_{O_{i}},y_{O_{i}}\right)$, and the articulated mechanism $\left(x_{J_{i}},y_{J_{i}}\right)$. The attitudes, yaw velocity of each vehicle element, the steering angle and velocity of each wheel which recorded separately as *φ*_*i*_, *ω*_*i*_, *δ*_*ijk*_ and *v*_*ijk*_ are all included. The mapping function which is labelled as Γ from the planned path to these physical quantities which control the motion features of the vehicle could be written as following.

$$\left\{\left({}_{O_{i}},y_{O_{i}}\right),\varphi_{i},\omega_{i},\delta_{ijk},{\vec{v}}_{J_{i}}(x_{J_{i}},y_{J_{i}})\right\}=\Gamma \left\{g(x),{\vec{v}}_{O_{bm}},(x_{O_{bm}},y_{O_{bm}})\right\}$$ (8)

In the equation, *g*(*x*) is the planned path, and ${\vec{v}}_{O_{bm}}$ is the velocity of each trace point. Parameter *m* is the number of trace points. Parameter *i* is the number of vehicle elements. Parameter *j* and *k* represent the location of each wheel.

### 3.1. Boundary constraint conditions

The essence of path planning of the vehicle is the problem of curve generation from given original state and the terminal state. The generated curve should meet the two requirements listed below. The cycle of path planning begins from the original state and ends at the terminal state. The start and end time of the cycle are labelled as *t*_*b*_ and *t*_*e*_. ***C***_***b***_ and ***C***_***e***_ represent the physical quantities of the original and terminal motion state which could be assigned as Eqs (9) and (10).

$$C_{b}=\{(x_{bm},y_{bm}),\varphi_{bi},\delta_{bijk},d_{bij},{\vec{v}}_{bijk},\omega_{bijk}\}$$ (9)

$$C_{e}=\{(x_{em},y_{em}),\varphi_{ei},\delta_{eijk},d_{eij},{\vec{v}}_{eijk},\omega_{eijk}\}$$ (10)

The constraints of the original and terminal boundary are expressed as the control of the location and shape of the generated curve. The trace points should be always on the curve and the tangent slope of each trace point on the curve is given by the boundary constraint. The constraint equation is shown in Eq (11).


$$\{\begin{array}{l} y_{O_{bm}}=g(x_{O_{bm}})\\ \frac{v_{yO_{bm}}}{v_{x}O_{bm}}=g^{\prime }(x_{O_{bm}})\\ y_{O_{em}}=g(x_{O_{em}})\\ \frac{v_{yO_{em}}}{v_{xO_{em}}}=g^{\prime }(x_{O_{em}}) \end{array}$$ (11)

### 3.2. Curve generation method

Cubic spline and B-Spline interpolation are appropriately considered the kinematic characteristics of the vehicle. The two curves could fit the function values in trace points Which are both second order derivatives to control the radius of curvature. These advantages are good to control the continuity of the path. However, the first-order derivative is uncontrollable which means the direction of the curve is uncertain. The higher-degree polynomial is all right to control both the value and the first-order derivative in trace points, but the curvature of planned curve is hard to guarantee. Thus, a method of segmental interpolation based on quartic polynomial is proposed here.

The polynomial equation in each segment is expressed as Eq (12).

$$P_{h}(x)=\sum_{j=0}^{4}a_{hj}x^{j}$$ (12)

Thus, the first and second order derivatives are expressed as Eqs (13) and (14).

$${P^{\prime }}_{h}(x)=\sum_{j=1}^{4}j\cdot a_{hj}x^{j-1}$$ (13)

$${{P^{\prime }}^{\prime }}_{h}(x)=\sum_{j=2}^{4}j\cdot (j-1)\cdot a_{hj}x^{j-2}$$ (14)

The function value and the first derivative of each trace point are given by the boundary constraint Eq (11). Bring them into (13) and (14), we can get the following equation.

$$y_{bm}=P_{h}(x_{bm})=\sum_{j=0}^{4}a_{hj}{\left(x_{bm}\right)}^{j}$$ (15)

$$y_{em}=P_{h}(x_{em})=\sum_{j=0}^{4}a_{hj}{\left(x_{em}\right)}^{j}$$ (16)

$$k_{em}={P^{\prime }}_{h}(x_{em})=\sum_{j=1}^{4}j\cdot a_{hj}{\left(x_{em}\right)}^{j-1}$$ (17)

$$k_{bm}={P^{\prime }}_{h}(x_{bm})=\sum_{j=1}^{4}j\cdot a_{hj}{\left(x_{bm}\right)}^{j-1}$$ (18)

The characteristics of the segmental interpolation based on quartic polynomial should guarantee the continuity of function values and the second order derivatives in the boundary points. Thus, we can get,

$$P_{h-1}(x_{m}{}^{-})=P_{h}(x_{m}{}^{+}),\;\sum_{j=0}^{4}a_{(h-1)j}x_{m}{}^{j}=\sum_{j=0}^{4}a_{hj}x_{m}{}^{j}$$ (19)

$${P^{\prime }}_{h-1}(x_{m}{}^{-})={P^{\prime }}_{h}(x_{m}{}^{+}),\;\sum_{j=0}^{4}j\cdot a_{(h-1)j}x_{m}{}^{j-1}=\sum_{j=0}^{4}j\cdot a_{hj}x_{m}{}^{j-1}\;(h\geq 2)$$ (20)

$${{P^{\prime }}^{\prime }}_{h-1}(x_{m}{}^{-})={{P^{\prime }}^{\prime }}_{h}(x_{m}{}^{+}),\;\sum_{j=2}^{4}j\cdot (j-1)\cdot a_{(h-1)j}x_{m}{}^{j-2}=\sum_{j=2}^{4}j\cdot (j-1)\cdot a_{hj}x_{m}{}^{j-2}\;(h\geq 2)$$ (21)

If ***n*** represents the number of interpolating points, the number of the equations listed above is 5*n*-6 and the number of undetermined coefficients of the piecewise curve based on quartic polynomial is 5*n*-5. The curvilinear equation could be uniquely determined when any equation is given. In general, the radius of curvature in the last trace point of the terminal state is used here, which is expressed as Eq (22).

r(xmmax)=[1−P′h(xmmax)]32P′′h(xmmax) (22)

The expression of the planned feasible path of the vehicle in the cycle is determined uniformly according to these simultaneous equations. The physical quantities which describe the motion could also be calculated to guarantee the vehicle to move along the planned path.

## 4. The circulation of feasible path planning

According to the kinematics model of the vehicle, the values of the variables which control the motion of vehicle are related to the actual operating path when the location and quantities of the trace points are confirmed. Thus, the coordinate trajectory tracking strategy of multi-units of the whole vehicle is converted to the planning of feasible operating path. When the detected tracking error is larger than the preset virtual track, a new preview point is selected from the virtual track as the terminal boundary of circulation. The feasible operating path is preplanned and the values of control variables are recalculated. The decision condition of the circulation and the selection method of the preview point are described in detail.

### 4.1. Circulation condition

**Fig 5:**
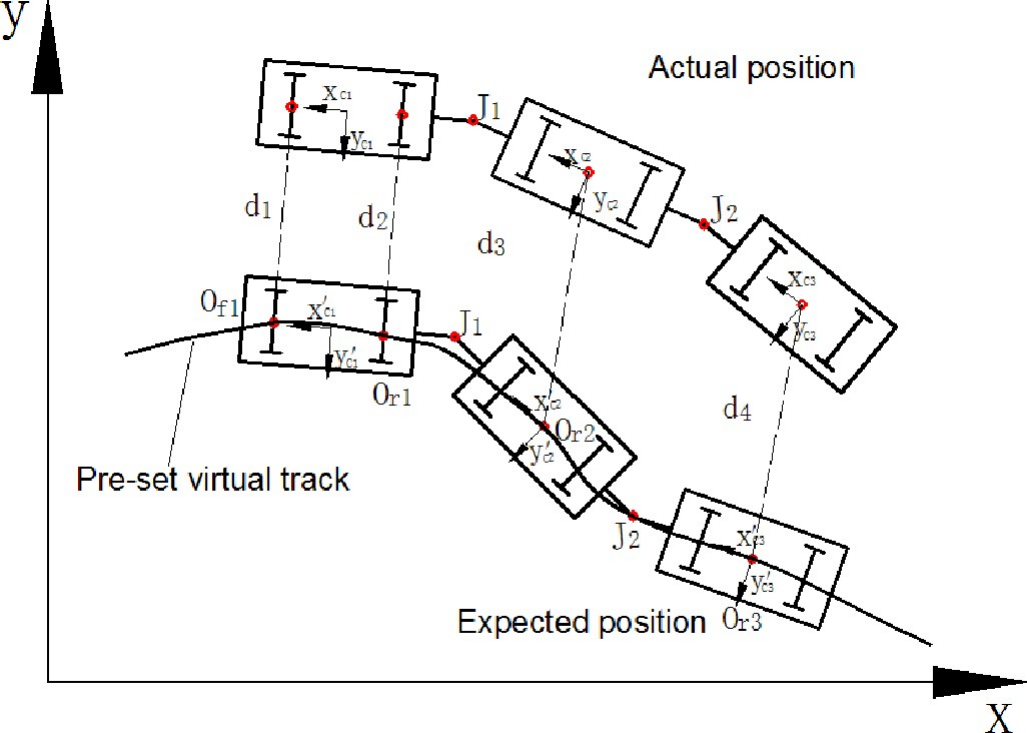
The tracking error of the whole vehicle.


The tracking error of the whole vehicle should be defined first. There are two essential means to represent the tracking error. The first is the distance from a certain point on the vehicle to a certain point on the virtual track, and the second is the difference between the actual attitude of the vehicle and the ideal one calculated by the virtual track. Thus, the tracking error of each vehicle unit should be considered. As is shown in Fig 5, the actual position of trace points is expressed as Eq (23) and the actual attitude of each vehicle element is labeled as *φ*_*i*_.

$$X_{B}=\left[\left(x_{O_{bm}},y_{O_{bm}}\right)\right]$$ (23)

The point with the minimum distance from the first trace point in the first vehicle element to the virtual track in the virtual track is regarded as the expected position of the first trace point. The expected positions and attitudes of other vehicle elements could also be calculated based on the method described above. The expected position of trace points is expressed as Eq (24). and the expected attitude of each vehicle element is labeled as ${\varphi^{\prime }}_{i}$.

$${X^{\prime }}_{B}=\left[\left({x^{\prime }}_{O_{bm}},{y^{\prime }}_{O_{bm}}\right)\right]$$ (24)

The total tracing distance error and attitude error of the vehicle could be expressed as the sum of the distance error of all trace points as Eqs (25) and (26).

$$d=\sum_{m=1}^{4}\left(d_{O_{bm}}\right)$$ (25)

$$\Delta \varphi =\sum_{i=1}^{3}\left(\left|\varphi_{i}\right|\right)$$ (26)

The upper limits of the distance error and attitude error are given as the threshold value for the circulation, which is labeled as *d*_max_ and Δ*φ*_max_. The decision condition of the circulation could be expressed as Eq (27).


$$\left(d=\sum_{m=1}^{4}\left(d_{O_{bm}}\right)\geq d_{\max}\right)\vee \left(\Delta \varphi =\sum_{i=1}^{3}(\left|\varphi_{i}\right|)\geq \Delta \varphi_{\max}\right)=1$$ (27)

### 4.2. Terminal boundary

At present, the preview distance is calculated by the product of vehicle speed and preview time.

L=vt (28)

As Eq (28) shows, the preview time here is an empirical value from 0.8 to 1.2. The quality of planned path could be evaluated by the curvature and its change rate. As Eq (29) shows, the index *k*_*t*_ is defined to evaluate the quality.

$$k_{t}=k_{1}{\left(r_{\min,\text{t}}-r_{\min}\right)}^{2}+k_{2}\frac{1}{{\left({\rho^{\prime }}_{\max,\text{t}}-{\rho^{\prime }}_{\max}\right)}^{2}}\;t=(0.8∼1.2)$$ (29)

The bigger the index ***k***_*t*_ is, the better quality the planned path possesses. In the equation, ***r***_min_ and ${\rho^{\prime }}_{\max}$ are the limit radius and change rate restrained by the characteristic of the vehicle.

## 5. Dynamics model of the unmanned vehicle

The degrees of freedom in dynamics model are selected according to the research purpose. The contents here are focused on path-following control of the whole guided bus. Thus, the response speed needs to be fast. The requirement of calculation speed and accuracy should be considered comprehensively. The most relative eleven degrees of freedom are built based on the following assumptions.

1. Only the planar motion paralleled of the pavement is considered;
2. The axle load transfer caused by the horizontal and longitudinal acceleration is considered;
3. The calculation difference of unspring mass is ignored.

**Fig 6:**
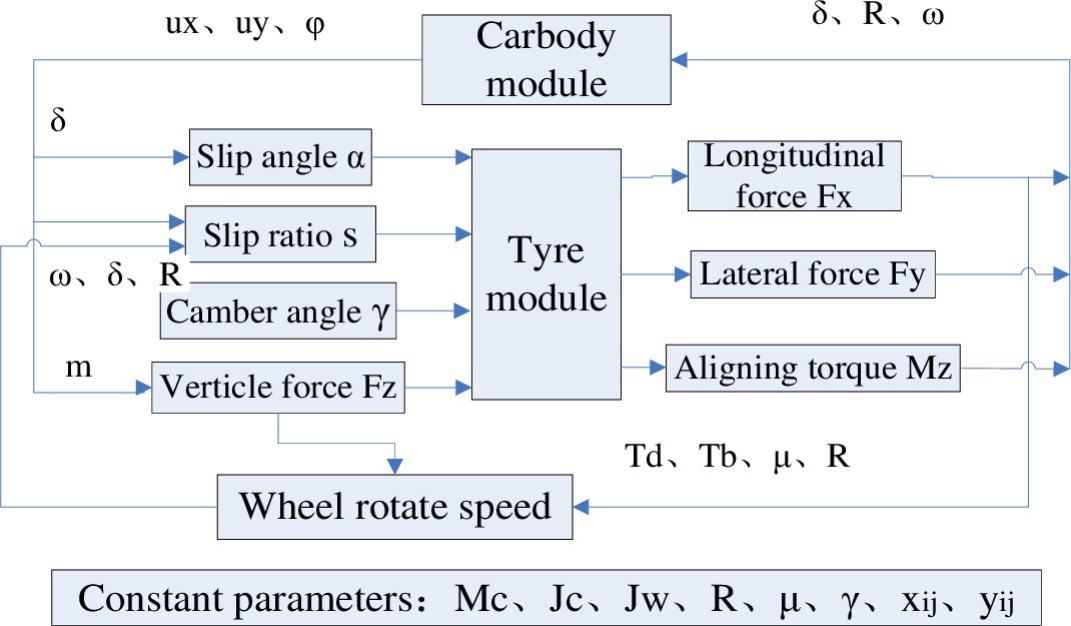
The main system modules of vehicle model.

The dynamic model of each vehicle element with the characteristics of all-wheel drived are built based on function modularization method. The main system modules include carbody module, tyre module, wheel hub motor module and a few correspondent modules shown in Fig 6. The required intermediate quantities such as slip angle, slip rate and wheel vertical forces are calculated according to the demand of each function modularization. The parameters used in the simulation such as the mass, rotational inertia and length of each body, tyre radius, friction coefficient, camber angle and other designation parameters are provided in designation.

As the steering angle of the left and right wheel is functioned by the same steering trapezium mechanism, there are nine independent degrees of freedom in total.

### 5.1. Articulated mechanism

**Fig 7:**
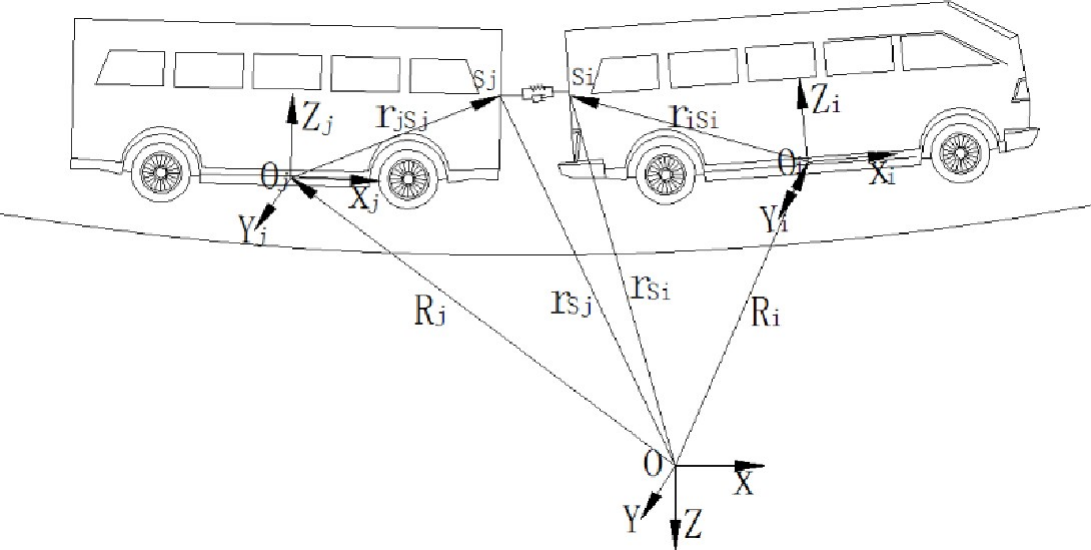
Spring-damper system.

As is shown in Fig 7, the articulated mechanism is constituted of kinematic pairs and force elements. The articulated mechanism should satisfy the needs of connection force and the freedom of motion between each vehicle element. Taking the spring-damper system as an example, the mechanical properties of the articulated mechanism are analyzed.

The function of spring-damper system here is used to decrease the impact of longitudinal impulse between each vehicle. The acting points of the force element system in the front and rear vehicle element are recorded as *S*_*i*_ and *S*_*j*_. As is shown in Eq (30), the distance vector $r_{S_{i}S_{j}}$ between *S*_*i*_ and *S*_*j*_ could be calculated by the position vector.

$$r_{S_{i}S_{j}}=R_{i}+A_{i}{\bar{r}}_{iS_{i}}-(R_{j}+A_{j}{\bar{r}}_{jS_{j}})$$ (30)

$${\dot{r}}_{S_{i}S_{j}}=R_{i}+A_{i}{\bar{\bar{r}}}_{S_{i}}\bar{G}{\dot{\theta }}_{i}-(R_{j}+A_{j}{\bar{\bar{r}}}_{jS_{j}}\bar{G}{\dot{\theta }}_{j})$$ (31)

In the equation, ${\dot{r}}_{S_{i}S_{j}}$ is the velocity vector and ${\dot{\theta }}_{j}$ is the first-order derivative of the attitude in the generalized coordinate. Thus, the acting force ***F***_*S*_ could be expressed as Eq (32).

$$F_{S}=K(r_{S_{i}S_{j}}-r_{S_{i}S_{j}}^{0})+C\left({\dot{r}}_{S_{i}S_{j}}\right)$$ (32)

***K*** and ***C*** are the stiffness matrix and the damping matrix separately. $r_{S_{i}S_{j}}^{0}$ is the initial distance vector. The form of generalized force vector could be described as Eq (33).


$$Q_{i}=-Q_{j}=\left[\begin{array}{c} F_{S}e_{q}\\ -\bar{G^{T}}A_{i}^{T}{\bar{\bar{r^{T}}}}_{S_{i}}F_{S}e_{q} \end{array}\right]$$ (33)

### 5.2. Dynamics model of the unmanned articulated vehicle

The vehicle should consider the influence of the connection between each conjoint element. In the modeling, each vehicle element is regarded as a basic unit which is the same with the dynamic model above. Then the connection forces are considered. The modeling method of long-large train based on the loop variable developed by CHI [14] is used here. The basic idea of this method is to regard each vehicle element as central integral unit. The kinematic equation could be expressed as

$$M\ddot{X}+C\dot{X}+KX=P$$ (34)

In which, ***M***, ***C*** and ***K*** represent the mass matrix, damping matrix and stiffness matrix of the vehicle element. $\ddot{X}$, $\dot{X}$ and ***X*** are the generalized acceleration vector, velocity vector and displacement vector. ***P*** is the generalized load vector.

The Eq (35) changes to (36) while considering the acting forces ***F*** between each vehicle element.

$$M\ddot{X}+C\dot{X}+KX=P+F$$ (35)

The Eq (35) is expanded for each basic integral unit, which express as follow.

$$M_{i}{\ddot{X}}_{i}+C_{i}{\dot{X}}_{i}+K_{i}X_{i}=P_{i}+F_{i}$$ (36)

**Fig 8:**
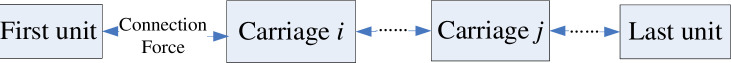
The calculation method of long-large train based on the loop variable.

In this way, the integral of the whole vehicle is divided into small integral units as Fig 8 shows. While giving the initial value and acting force of each vehicle element, the dynamic models of the whole vehicle could be built.

## 6. Verification of the trajectory tracking strategy

The coordinate trajectory tracking strategy of the vehicle based on the circulation of feasible path planning was verified by the constructed simulation platform. The simulation platform was built based on the dynamics model and trajectory calculation model. The values of control variables were calculated according to the re-planned path and then transferred to the dynamics model. The actual motion trajectory and the kinetic parameters were calculated. The operating velocity was controlled by the first trace point which is preset at a medium constant value.

The trajectory of lemniscate was preset as the virtual track. This kind of track was always used in vehicle handling and stability testing. It was suitable to verify the trajectory tracking ability of the vehicle considering the executability and stability. The equation of the preset track was shown in Eq (37).

$$l-60*\sqrt{\cos(2\psi )}$$ (37)

**Fig 9:**
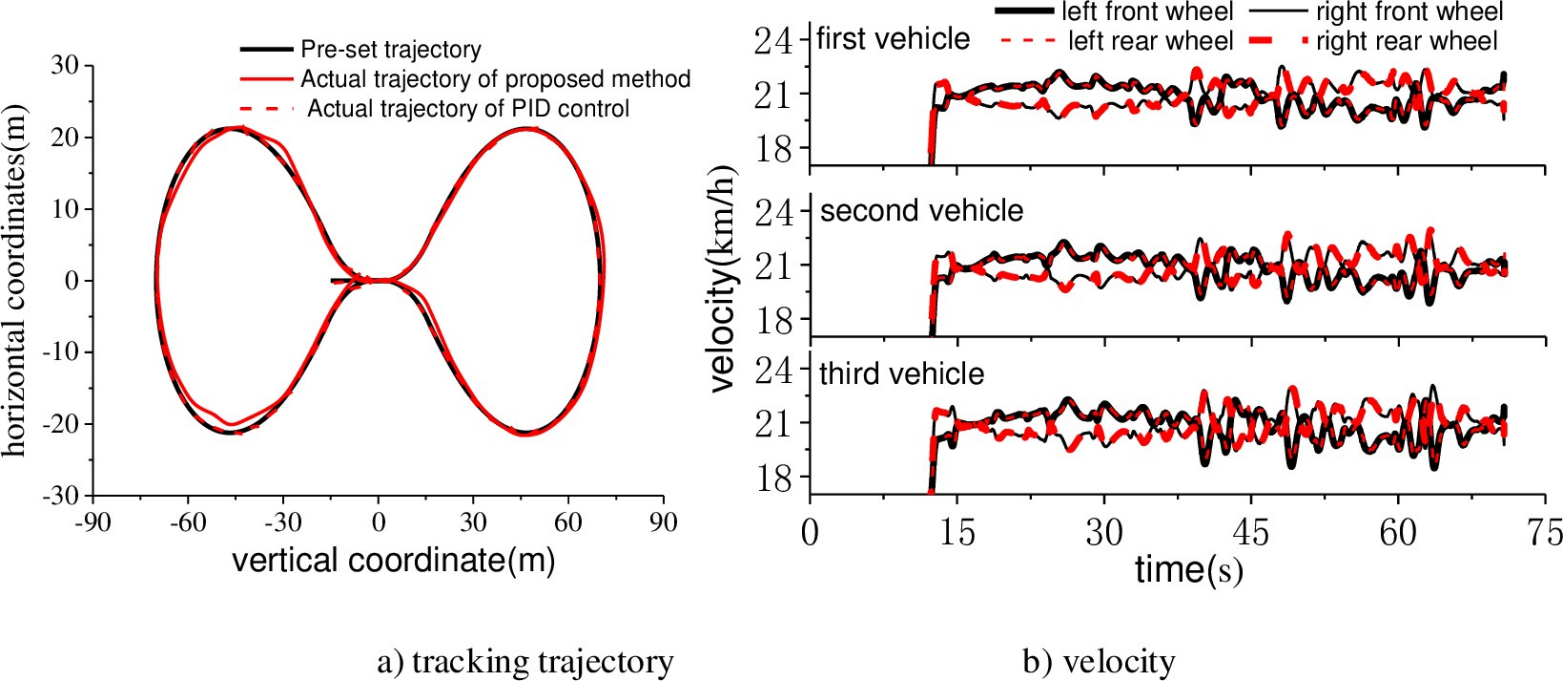
The comparison of pre-set and motion trajectory and the velocity of each wheel.

The tracking effect of the control method proposed in this paper was compared with the traditional PID control method. As was shown in Fig 9, the solid black line in the figure was the preset lemniscate trajectory. The red solid line was the actual trajectory of the tracking method proposed in this paper. The red dotted line was the actual trajectory of PID method. Results showed that, the overall tracking error of PID control was slightly better than the tracking method proposed in this paper. But the overshoot phenomenon appears a few times, which directly reflected in the decrease of vehicle stability, especially for multiple articulated vehicles. The tracking error of the algorithm proposed in this paper was larger than that of PID control as a result of considering the kinematic coordination of multiple carriages. The tracking accuracy was reduced slightly as the cost of improving the overall stability of the vehicle.

The controller had detected sixteen times of the condition that the position or attitude error exceeded the preset threshold value. Under the circumstances, the controller conducted the command of feasible path re-planning to adjust the position and attitude to follow the guidance in front of the virtual track. The speed of the first trace point was constant at 6 m/s. The actual speed of each wheel controlled by hub motors was exported as Fig 9. The fluctuation of the velocity curves was agreed with the time of path re-planning which mean that the hub motors execute as control instructions. The vehicle moved at the right side of the lemniscate first. The processes of path re-planning for trajectory tracking were increased obviously. As a result, the velocity curves and the actual trajectory of the vehicle were unsmooth than that of the left side. The whole variant trend of the velocity curves of each vehicle was nearly the same. However, there was a difference of time phase to guarantee each vehicle element to path through the planned feasible curve in turns.

**Fig 10:**
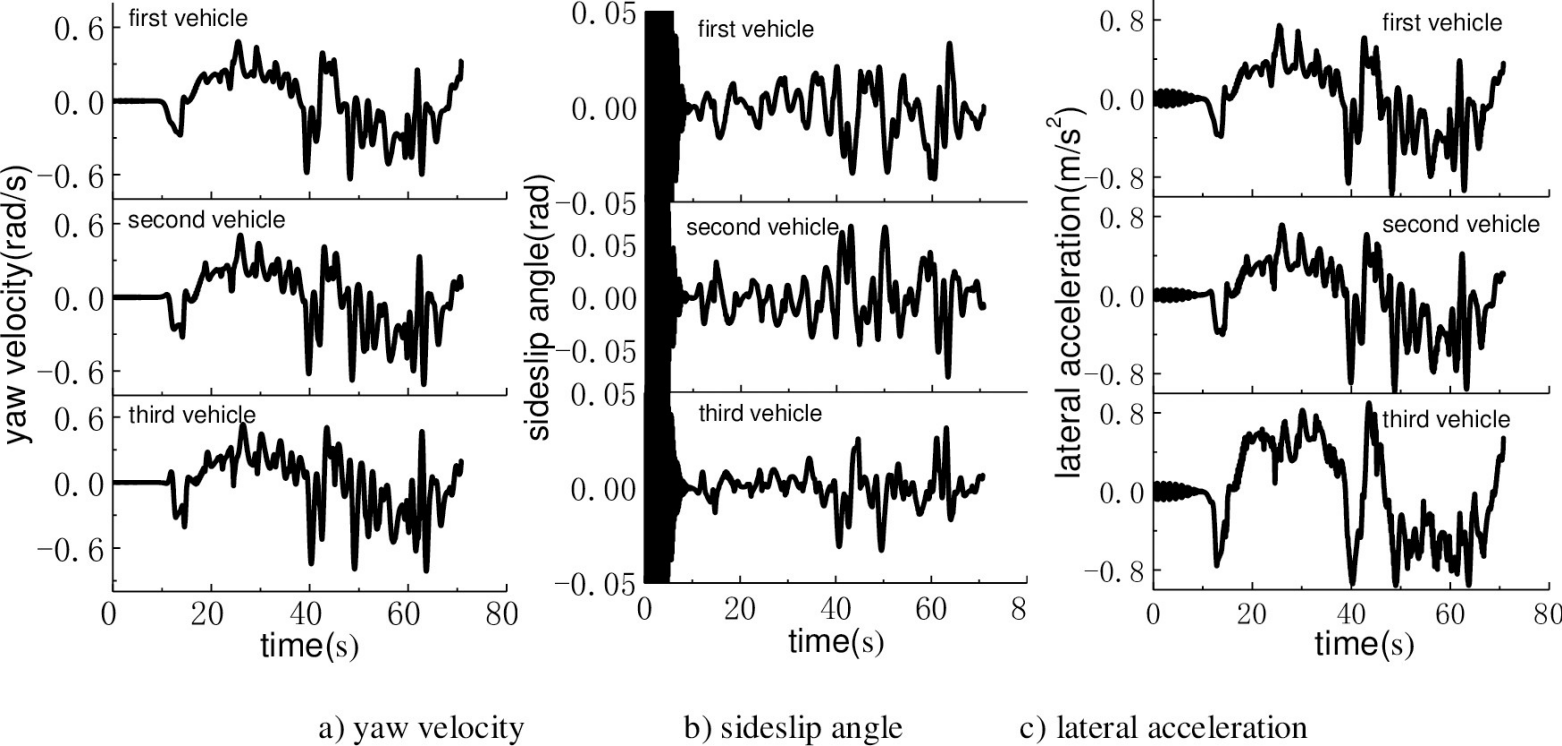
The dynamics performances in the tracking process.

The dynamics performances in the tracking process were plotted as Fig 10. The yaw rate, side slip angle and lateral acceleration were selected as quantified indicators. The three dynamics indicators were all fluctuated as the process of path re-planning. It was preferable that the dynamics indicators were in the normal range in the trajectory tracking process. Simulated results indicated that the proposed method was of good trajectory tracking control ability.

## 7. Conclusions

A new kind of trajectory tracking method based on the circulation of feasible path planning for the unmanned vehicles were proposed in this article. As the core technology of the unmanned vehicle, the feasible path planning method based on the kinematics model were analyzed. The expected position determination method of the unmanned vehicles was proposed first to locate the vehicle. Then, the boundary constraint conditions were analyzed, and curve generation method was proposed to generate feasible path of the whole vehicle. Finally, the trajectory tracking based on the circulation of feasible path planning was proposed. The Circulation condition and terminal boundary of the circulation were analyzed. The dynamics model of the unmanned vehicle was built to reflect its real service status and verify the trajectory tracking strategy. The results showed that the coordinate traction control strategy of the vehicle based on the circulation of feasible path planning was fairly good in the preset lemniscate track.

## Supporting information

S1 Data (XLSX)
